# Selective Concurrence of the Long Non-Coding RNA MALAT1 and the Polycomb Repressive Complex 2 to Promoter Regions of Active Genes in MCF7 Breast Cancer Cells

**DOI:** 10.3390/cimb45060301

**Published:** 2023-05-30

**Authors:** Felipe Arratia, Cristopher Fierro, Alejandro Blanco, Sebastian Fuentes, Daniela Nahuelquen, Martin Montecino, Adriana Rojas, Rodrigo Aguilar

**Affiliations:** 1 Institute of Biomedical Sciences (ICB), Faculty of Medicine and Faculty of Life Sciences, Universidad Andres Bello, Santiago 8370071, Chile; 2 Institute of Human Genetics, Faculty of Medicine, Pontificia Universidad Javeriana, Bogotá 110211, Colombia

**Keywords:** long non-coding RNA, MALAT1, Polycomb Repressive Complex 2, chromatin

## Abstract

In cancer cells, the long non-coding RNA (lncRNA) MALAT1 has arisen as a key partner for the Polycomb Repressive Complex 2 (PRC2), an epigenetic modifier. However, it is unknown whether this partnership occurs genome-wide at the chromatin level, as most of the studies focus on single genes that are usually repressed. Due to the genomic binding properties of both macromolecules, we wondered whether there are binding sites shared by PRC2 and MALAT1. Using public genome-binding datasets for PRC2 and MALAT1 derived from independent ChIP- and CHART-seq experiments performed with the breast cancer cell line MCF7, we searched for regions containing PRC2 and MALAT1 overlapping peaks. Peak calls for each molecule were performed using MACS2 and then overlapping peaks were identified by bedtools intersect. Using this approach, we identified 1293 genomic sites where PRC2 and MALAT1 concur. Interestingly, 54.75% of those sites are within gene promoter regions (<3000 bases from the TSS). These analyses were also linked with the transcription profiles of MCF7 cells, obtained from public RNA-seq data. Hence, it is suggested that MALAT1 and PRC2 can concomitantly bind to promoters of actively-transcribed genes in MCF7 cells. Gene ontology analyses revealed an enrichment of genes related to categories including cancer malignancy and epigenetic regulation. Thus, by re-visiting occupancy and transcriptomic data, we identified a key gene subset controlled by the collaboration of MALAT1 and PRC2.

## 1. Introduction

The Polycomb Repressive Complex 2 (PRC2) is an epigenetic modifier that catalyzes the methylation of lysine 27 on histone H3 [1]. The presence of the complex and its mark in gene regulatory regions often leads to gene repression [2]. We and others have shown that such repression is required for successful cellular differentiation and development [1,3]. However, this complex has also been shown to bind to nascent transcripts in transcribing genes [4]. PRC2 is constituted of a core of at least three proteins: EZH2 (the catalytic subunit, which can be replaced by EZH1 in postmitotic cells such as neurons [5]), SUZ12 and EED, plus additional accessory subunits [1]. The misregulation of PRC2 is found in several types of cancer cells, including breast cancer, contributing to the nonmutational epigenetic reprogramming that facilitates the acquisition and maintenance of malignancy [6]. Currently, drugs targeting different sub-units of PRC2 are under development as cancer treatment alternatives [7].

In the epigenetics field, how PRC2 is targeted to specific genes is still a matter of active discussion [8]. Since the Polycomb response elements originally identified in *Drosophila* are not conserved in mammalian genomes, different research groups have unsuccessfully attempted to uncover DNA methylation- or protein-dependent mechanisms that apply to the whole genome. Two seminal papers from the late 2000s reported that the long non-coding RNAs (lncRNAs) Hotair [9] and Xist [10] were sufficient for recruiting PRC2 to the *HOX* locus and the inactive X chromosome, respectively, portraying lncRNA-PRC2 interactions as a paradigm of molecular collaboration, at least at those specific genomic regions. Recently, the Xist-PRC2 interaction was specifically disrupted by novel small molecules designed to target the lncRNA [11].

Another PRC2-interacting lncRNA is MALAT1. This transcript has been under the spotlight of cancer biologists since its discovery in 2003 in small lung cancer cells [12]. In subsequent studies, MALAT1 was found in several additional types of tumors, including breast cancer, correlating with poor prognosis. Thus, MALAT1 has arisen as an archetype of lncRNA function in malignancies [13,14]. As for PRC2, MALAT1 overexpression has been proposed as a cancer biomarker [15], and it currently represents a promising therapeutic target to treat tumor progression [16].

There is increasing evidence indicating that MALAT1 partners with chromatin-modifying enzymes, including the histone deacetylase HDAC9, the chromatin remodeler BRG1, or the RNA modifier METTL16 [17,18]. PRC2 is, however, one of the most studied MALAT1 partners in tumors [19]: first, RNA immunoprecipitation (RIP) experiments performed on cancer cell extracts show that both molecules are part of a complex in the nucleoplasm [20,21,22]; second, genes such as PCDH10 and E-cadherin are derepressed in cancer cells following MALAT1 or PRC2 down-regulation [21,22,23]. Additional works show that the MALAT1–PRC2 interaction seems to be dependent on the 3′-end of the lncRNA in cancer cells [23], but whether other complexes follow similar recruitment mechanisms is unclear.

One key question that remains to be addressed in cancer cells is whether the MALAT1–PRC2 partnership occurs in chromatin at a genome-wide level and whether this necessarily results in gene repression. Since the PRC2 subunit EZH2 can bind and regulate target genes as a single protein by histone methylation-independent mechanisms [24], experiments focusing on the chromatin-binding profile of the core subunit SUZ12 are better suited for studying the interaction sites of the full PRC2 [25]. Unlike chromatin immunoprecipitation (ChIP), which is the gold standard for the study of protein–DNA binding in vivo [26], examples of lncRNA-capture experiments (CHART, ChIRP or RAP) to define genomic binding sites for lncRNAs are scarce in the literature and databases. This is due to the challenge of capturing specific RNA molecules and performing the deep sequencing of the DNA associated with them [27,28,29,30]. Hence, up to the present date, reports claiming that a MALAT1–PRC2 complex modulates the expression of key target genes in cancer cells are based on the assumption that MALAT1 binds to that particular DNA sequence (e.g., [22]). Moreover, only a few authors have performed the experiments required to show the global MALAT1 binding profile in humans [28] or mice [27,29,30].

Here, we revisit two genome-wide sequencing studies performed by independent laboratories providing a novel perspective: we unified a previous report that used CHART to identify MALAT1 binding sites in MCF7 breast cancer cells [28] with an independent study performed in the same cell line evaluating the binding of the SUZ12 subunit of PRC2 to the genome [31]. We searched in silico for genes associated with overlapped peaks of PRC2 and MALAT1, aiming to identify a set of genes and main Gene Ontology categories, where MALAT1-PRC2 may collaborate to regulate transcription in MCF7 cells. Our results indicate that a number of genes bound by MALAT1–PRC2 are enriched in the GO categories of cancer malignancy and epigenetic regulation, and they are mostly actively transcribed in MCF7 breast cancer cells.

## 2. Materials and Methods

### 2.1. CHIP- and CHART-Seq Datasets

The original MALAT1 CHART-seq experiments were performed by the Robert Kingston lab [28] in the MCF7 breast cancer cell line [32] and the raw sequencing data were deposited as Sequence Read Archives (SRA) that were recovered using the following run numbers: Replica 1: SRR1386233 (https://www.ncbi.nlm.nih.gov/geo/query/acc.cgi?acc=GSM1411209 (accessed on 1 July 2021) [33]); Replica 2: SRR1386234 (https://www.ncbi.nlm.nih.gov/geo/query/acc.cgi?acc=GSM1411210 (accessed on 1 July 2021) [34]); Input: SRR1386236 (https://www.ncbi.nlm.nih.gov/geo/query/acc.cgi?acc=GSM1411212 (accessed on 1 July 2021) [35]). The SUZ12 ChIP-seq experiments were performed by the Michael Snyder lab and the raw SRA were recovered from ENCODE [31] using the following run numbers: Replica 1: SRR6214195 (https://www.ncbi.nlm.nih.gov/geo/query/acc.cgi?acc=GSM2828862 (accessed on 1 July 2021) [36]); replica 2: SRR6214196 (https://www.ncbi.nlm.nih.gov/geo/query/acc.cgi?acc=GSM2828863 (accessed on 1 July 2021) [37]), input: SRR5111271 (https://www.ncbi.nlm.nih.gov/geo/query/acc.cgi?acc=GSM2423179 (accessed on 1 July 2021) [38]).

### 2.2. Quality Control, Alignment and Peak Calling

Since MALAT1 and SUZ12 are broadly distributed throughout the genome [27,28], the fastq sequencing archives were subjected to the ENCODE 3 histone ChIP-seq pipeline in histone (non-transcription factor) mode (https://github.com/ENCODE-DCC/chip-seq-pipeline2 (accessed on 1 July 2021)) [39]; see also [40]. Briefly, fastq sequence files derived from SRAs were cropped using Trimmomatic v0.39 [41]. Reads were aligned against the human hg38 genome using Bowtie2 v2.2.6 [42] with paired-end ChIP-seq parameters. Unmapped reads, multi-mapped reads, and duplicates were removed using MarkDuplicates in Picard v1.126 (http://broadinstitute.github.io/picard/ (accessed on 1 July 2021)) [43]. Peak calling was performed using MACS2 [44] in paired-end mode, selecting the optimal peak set for subsequent experiments. Bed and subsequent Bigwig files were loaded into the Integrative Genomics Viewer (IGV) v2.8 or above to visualize peaks and signals.

### 2.3. Detection of Overlapped Peaks and Gene Ontology Analyses

To identify regions of the genome where MALAT1 and SUZ12 peaks overlap in at least 1 nucleotide, optimal peak bed files were analyzed with bedtools intersect v2.29.2 or higher (https://bedtools.readthedocs.io/en/latest/content/tools/intersect.html (accessed on 1 July 2021)) [45] using the default parameters (“bedtools intersect -a malat1_peaks.bed -b suz12_peaks.bed >> intersect_malat1_vs _suz12.bed”. Peak annotation was performed using the R package ChIPseeker v3.10 or higher (https://bioconductor.org/packages/release/bioc/html/ChIPseeker.html (accessed on 1 July 2021)) [46] using “flankDistance = 5000”. The nearest genes around peaks were analyzed using the gene ontology enrichment analysis tool from PANTHER Classification System v16.0 or higher (http://pantherdb.org/ (accessed on 1 July 2021)) [47].

### 2.4. RNA-Seq Analyses

Two independent transcriptomic analyses of MCF7 cells were performed from raw sequencing data deposited as SRAs that were recovered using the following numbers: SRR2749729 (https://www.ncbi.nlm.nih.gov/geo/query/acc.cgi?acc=GSM1915041 (accessed on 1 November 2022) [48]) [49] and SRR925723 (https://www.ncbi.nlm.nih.gov/geo/query/acc.cgi?acc=GSM1172885 (accessed on 1 November 2022) [50]) [51]. Transcript level abundance was estimated using Salmon v1.9 (https://salmon.readthedocs.io/en/latest/ (accessed on 1 November 2022)) [52] and expressed as Transcripts per Million (TPM). Transcripts were aggregated to the gene level using the R package tximport against the human transcriptome (https://bioconductor.org/packages/release/bioc/html/tximport.html (accessed on 1 November 2022)).

## 3. Results

Raw SRA sequence files from ChIP- [36,37,38] and CHART-seq [33,34,35] experiments were downloaded from the Gene Expression Omnibus and subjected to the ChIPseq processing pipeline recommended by ENCODE [39]. We confirmed that the deposited datasets were suitable for this analysis after performing quality control checks showing sufficient mapped reads (>50 million for SUZ12 and MALAT1 after filtering) with acceptable reproducibility among replicates (key features shown in Supplementary Table S1).

**Figure 1:**
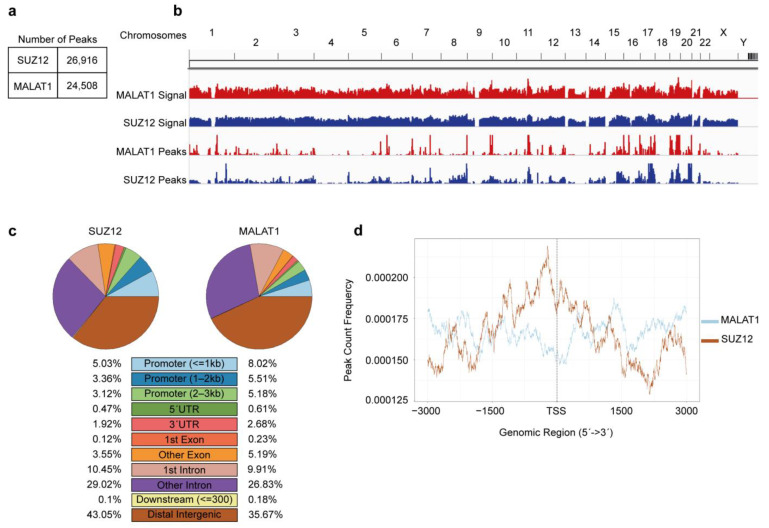
Genomic distribution of MALAT1 and SUZ12 in the MCF7 breast cancer cell line. (**a**) Raw sequencing data deposited in the Gene Expression Omnibus were subjected to the ChIPseq processing pipeline recommended by ENCODE. The number of reproducible peaks was determined for each molecule separately, as shown in the table. (**b**) Signal (bigwig) files and peak (bed) files were loaded into the Integrative Genomics Viewer. MALAT1 data are shown in red tracks and SUZ12 data in blue tracks. Chromosome numbers as shown on top of the graph. (**c**) Genomic annotations for optimal peaks were obtained using ChIPseeker. The percentage of peaks contained in each feature (colored rectangles) is shown for SUZ12 (left) and MALAT1 (right). (**d**) Metagene analyses were performed to evaluate the frequency of the peaks found around the transcription start site (TSS) for MALAT1 (blue line) and SUZ12 (brown line). Distance from the TSS in base pairs is shown in the x-axis.

We first analyzed the binding profile of each molecule separately. The number of optimal peaks (peaks consistently found in two replicates) was determined using an irreproducible discovery rate (IDR) value below 0.01 as filter. We identified 26,916 peaks for SUZ12 enrichment and 24,508 for MALAT1 (Figure 1a and Supplementary Table S1). These peaks were distributed throughout the human genome (Figure 1b) with no preference for promoter regions (Figure 1c). Instead, we found SUZ12 or MALAT1 preferentially located on introns and intergenic regions (Figure 1c). Additional metagene analyses revealed that, when focusing on a −3000/+3000 region (around TSSs), SUZ12 and MALAT1 showed a mutually exclusive enrichment pattern, with SUZ12 enriched over the TSS and MALAT1 voided from the same region (Figure 1d).

**Figure 2:**
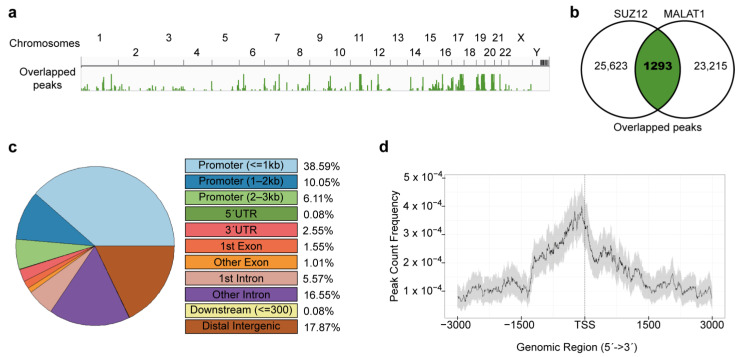
Genomic distribution of MALAT1-SUZ12 overlapped peaks in the MCF7 breast cancer cell line. (**a**) Reproducible peaks from SUZ12 and MALAT1 experiments were subjected to bedtools intersect to detect sites of overlap. The resulting bed file was loaded into the Integrative Genomics Viewer and is shown in green bars. Chromosome numbers as shown on top of the graph. (**b**) The number of peaks shared by SUZ12 and MALAT1 is shown with green background in the Venn diagram. (**c**) Genomic annotations for overlapped peaks were obtained using ChIPseeker. The percentage of peaks contained in each feature (colored rectangles) is shown. (**d**) Metagene analyses were performed to evaluate the frequency of the overlapped peaks (black line) found around the transcription start site (TSS) within a confidence interval of 95% (grey background). Distance from the TSS in base pairs is shown on the x-axis.

We next assessed whether SUZ12 and MALAT1 present overlapped enrichment peaks throughout the genome. To identify those overlapping peaks, we applied bedtools intersect to the BED files containing the optimal peaks of SUZ12 and MALAT1. We found 1293 shared peaks between both molecules (Figure 2a,b), associated with 839 genes (Supplementary Table S2). Metagene analyses showed that the overlap of SUZ12 and MALAT1 occurs preferentially in genomic regions where a number of gene promoters are located, as 708 peaks (54.75% of the total number of peaks, which are associated with 531 genes) reside within the −3000/+1 region (Figure 2c, and Supplementary Table S2), with a notorious enrichment around the TSS (Figure 2d).

**Figure 3:**
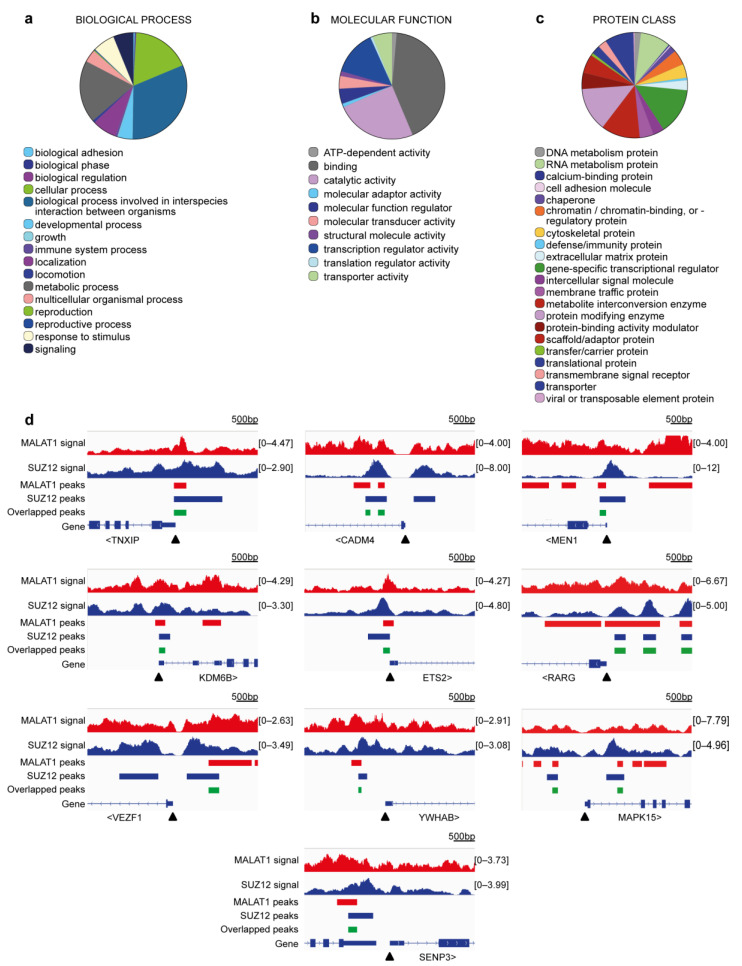
MALAT1 and SUZ12 concur to gene promoter regions of cancer-related genes. (**a**–**c**) Gene ontology analyses were performed in genes presenting MALAT1 and SUZ12 overlap within the 3000/+1 region. Categories for biological process (**a**), molecular function (**b**), and protein class (**c**) are shown. In (**d**) signal (bigwig) files and peak (bed) files were loaded into the Integrative Genomics Viewer. MALAT1 data are shown in red tracks, SUZ12 data in blue tracks, and overlap sites in green. Genes positions are shown, with arrowheads indicating the transcription start site. The fragments per million mapped fragments (FPM) scale is shown in brackets. Only one splice variant is shown for each gene. Scale bar: 500 bp.

We then focused our analysis on a group of 403 genes where MALAT1 and PRC2 overlap immediately upstream of the TSS (−1000/+1 region, Supplementary Table S2), as this co-localization has a higher potential for establishing mechanisms that regulate the expression of those target gene promoters [1,4]. Thus, a gene ontology (GO) analysis was carried out by loading the IDs of these genes into the PANTHER 17.0 platform. As depicted in Figure 3a–c, we determined an over-representation of genes associated with cancer-relevant functions, including biological adhesion, locomotion (Figure 3a), transcriptional regulation (Figure 3b), and chromatin binding (Figure 3c). In Table 1, we highlight 10 genes that, among other functions, have been found participating in signaling (*MEN1*, *YWHAB*, *SENP3*, *MAPK15*, and *CADM4*), oxidative stress (*TXNIP*), acting as transcription factors (*ETS2*, *RARG*, and *VEZF1*) or chromatin binders (*MEN1*, *KDM6B*) in cancer cells. The enrichment of MALAT1 (Figure 3d, red tracks) and SUZ12 (Figure 3d, blue tracks) was visualized using the Integrative Genomics Viewer software. In addition, the position of overlapped peaks around the TSS (Figure 3d, green rectangle) on each of the genes listed in Table 1, was marked.

**Figure 4:**
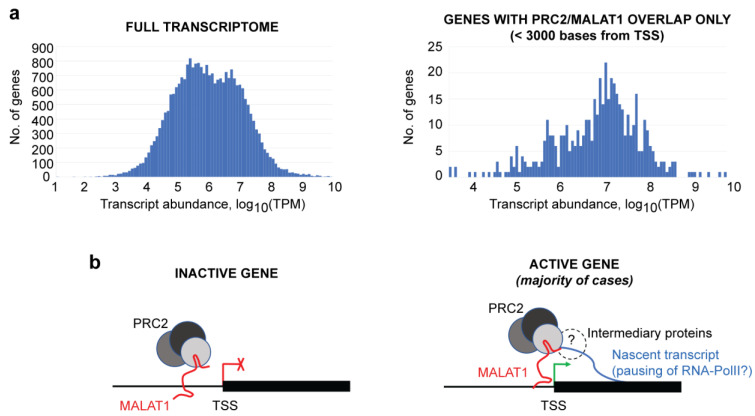
MALAT1 and SUZ12 concur to promoters of actively-transcribed genes. (**a**) transcriptomic abundance was determined from raw RNA sequencing files using Salmon and aggregated to the gene level. Left: frequency histogram showing all genes expressed in MCF7 cells with TPM (transcripts per kilobase million) > 0 derived from GSE74232/GSM1915041/SRR2749729 (total = 25,935 genes). Right: frequency histogram showing only the genes with MALAT1 and SUZ12 concurring to <3000 bp from TSS with TPM > 0 (total = 531 genes). (**b**) Scheme depicting MALAT1 and the PRC2 complex concurring to inactive (left) and active (right) genes. Our data indicate that, in most cases, MALAT1–PRC2 localize to active genes, where they may be interacting with nascent transcripts through unknown intermediary proteins (marked with “?”) (see Section 4).

Finally, we evaluated the transcriptional status of the genes where MALAT1 and PRC2 overlap. Raw SRA sequencing files from RNA-seq experiments were downloaded from the Gene Expression Omnibus and transcript abundances were obtained using Salmon and aggregated to the gene level. When 25,935 gene transcripts expressed in MCF7 cells were considered [48] (after filtering out genes with TPM values = 0), their abundances in one RNA-seq replicate [48] were distributed as shown in Figure 4a, left (1st quartile = 183,340 TPM; median = 1,016,602 TPM; 3rd quartile = 6,692,790 TPM; average = 24,485,358 TPM). Another independent RNA-seq study [50] showed similar distribution (Supplementary Table S3). We then selected the genes where MALAT1 and SUZ12 overlap within or near the gene promoter region (<3000 bp from the TSS), where we found that 487 of the 531 genes showing overlapping also exhibited significant expression levels (>0 TPM). Transcript abundances in one replicate are distributed as shown in Figure 4a, right (1st quartile = 1,930,667 TPM; median = 10,344,484 TPM; 3rd quartile = 30,084,933 TPM; average = 66,526,895 TPM). This distribution was reproducible in the two independent RNA-seq experiments that we analyzed (Supplementary Table S3). Together, these data indicate that MALAT1 and PRC2 can be preferentially co-localized at promoters of actively-transcribed genes.

## 4. Discussion

We present here an in silico study to identify genomic regions co-occupied by the lncRNA MALAT1 and the repressive epigenetic complex PRC2 in the MCF7 breast cancer cell line. We encountered a preferential concurrence of both macromolecules to genomic sequences near transcription start sites, often associated with actively-transcribed genes. A significant number of this set of target genes are related to signaling and gene expression control in cancer.

Since the discovery of MALAT1 in lung cancer cells [12], the epigenetic complex PRC2 has been proposed as a key regulatory partner [19], particularly after loss-of-function studies that measure the effect on specific target genes, where an independent knock-down of MALAT1 or of some of the PRC2 subunits results in gene de-repression [21,22,23]. The interaction of PRC2 and lncRNAs has been a matter of intense debate in the field, as some authors still consider the PRC2 catalytic subunit EZH2 as a promiscuous molecule, prone to bind to any RNA [63]. However, this idea has been challenged by several teams, proposing that PRC2–RNA complexes can collaborate in relevant regulatory mechanisms through specific interactions [64,65,66]. Therefore, there is a necessity for enlarging the number of studies that address, using multiple approaches, whether there is a binding of RNAs such as MALAT1 to DNA, narrowing the pool of genes where a potential PRC2–MALAT1 interaction may be mechanistically relevant.

We report that, despite the ubiquitous localization throughout the genome of both MALAT1 and the PRC2 subunit SUZ12, a clear overlap between the molecules is found only in a subset of 839 genes in MCF7 cells. There could be multiple mechanistic implications for this finding, and here we offer a few hypotheses that need further testing in the laboratory. First, these data support a model where MALAT1 may be involved in the recruitment of PRC2 to a subset of genes in breast cancer cells. This resembles the roles of Xist and Hotair [9,10] except that, unlike these, this is not confined to the X chromosome or to the *Hox* locus, but occurs in a widespread fashion. Importantly, the preferential concurrence of both molecules to regions upstream and near the transcription start site (TSS) of actively-transcribed genes captured our attention. As mentioned above, reports proposing a MALAT–-PRC2 complex focus almost exclusively on gene silencing [21,22,23], although this is in contrast to the seminal work demonstrating that MALAT1 is enriched on transcribed genes [28]. Together, these results may appear initially contradictory, but instead they argue in favor of a versatile role for RNA in general and for MALAT1 in particular, where these molecules can play a significant role during both gene activation and repression. Indeed, PRC2 can be found around the TSS, mediating at least two functions: H3K27me3 deposition, which leads to gene silencing [1] or *tempering* RNA Polymerase II elongation in actively-transcribed genes by binding to nascent transcripts [4,67]. The latter is further supported by a report showing that MALAT1 interacts with pre-mRNAs through RNA-binding proteins [29] which may include, according to our findings, PRC2 subunits.

Among the 403 genes that contain overlapping MALAT1–SUZ12 peaks at their proximal promoter regions (<1 kb from the TSS), special attention was given to genes involved in cancer progression, transcriptional regulation, and epigenetic activity. While the list presented in Table 1 is not exhaustive (the full gene list is available in Supplementary Table S2), it highlights interesting targets. Remarkably, all these genes are actively transcribing, exhibiting expression levels in the range of 1 to 10 million TPM in RNA-seq studies. First, two tumor suppressors caught our attention: the thioredoxin-interacting protein TXNIP and the cell adhesion molecule CADM4. Reduced levels of these two proteins have been linked to increased tumorigenesis and, accordingly, patients in higher cancer stages show low expression of both proteins [57,58]. Therefore, we are tempted to speculate that PRC2 is contributing to tempering their transcription, favoring breast cancer development. In the case of TXNIP, decreased levels prevent the clearance of oxidative species within the cells [58], while reduced levels of CADM4 limit the interaction with the extracellular matrix in cancer cells [57]. Menin and the lysine-demethylase KDM6B were also identified among the genes recognized by MALAT1 and SUZ12, and both are well-established epigenetic regulators. Menin is an essential component of the MLL/SET1 H3K4-methyltransferase complex, which is necessary for global gene activation, while KDM6B is an H3K27-demethylase, also regulating global gene transcription. In patients, low expression of menin favors tumorigenesis [53] and reduced levels of KDM6B favor the metastasis of breast cancer cells [62]. Additionally, we found that three transcription factor genes are targets of MALAT1-SUZ12: ETS2, RARG and VEZF1. ETS2 promotes telomerase expression [59], RARG has been linked with tamoxifen resistance in breast cancer patients [60], and VEZF1 is a regulator of angiogenesis [61]. In addition, we have highlighted the SENP3 gene, involved in protein sumoylation, whose elevated levels have been linked to poor survival in breast cancer patients [55]. Finally, genes participating in mitogen-activated pathways such as EGF and FGF, which are linked to cell growth and motility [54,56], are other genes that are targets of MALAT1 and SUZ12 binding. These genes encode the 14-3-3 protein YWHAB and the MAPK15 kinase. While YHWAB has a role in cell transformation [54], downregulation of MAPK15 (a proposed biomarker of breast cancer) increases cell motility in breast cancer and decreases apoptosis [56]. The potential involvement of a MALAT1–PRC2 complex in the regulation of genes connected to intricate regulatory networks (kinases, transcription factors, epigenetic regulators) explains why many authors have shown that loss-of-function experiments of MALAT1 or PRC2 subunits are sufficient to trigger phenotypic changes in cells. Additionally, our findings are an invitation not only to study the MALAT1–PRC2 pair from the perspective of H3K27me3-mediated gene silencing (which can be a minority of case, as depicted in Figure 4b, left), but also to increase the efforts in the study of mechanisms involving early transcription termination or RNA-Polymerase II pausing, where MALAT1-PRC2 may be acting as a rheostat that moderates transcription without suppressing it (Figure 4b, right) [4]. Biologists prone to taking a reductionist approach may find in these data and specific genes interesting models to conduct further experiments.

We are conscious that MCF7 is a human cell line representing a specific stage of a particular cancer. Nevertheless, it is also one of the most studied and characterized at the molecular level [68] and the only human cancer cell where RNA binding to the genome has been assayed using deep sequencing that is publicly available. Since PRC2-RNA-mediated regulation is tissue-specific [69], the patterns identified in this study may change in cells from different origins, including samples from healthy vs. diseased subjects. The patterns may also change among breast tumor cell lines representing different cancer stages where MALAT1 expression is modulated [70]. Recently, the Valerio Orlando team reported that MALAT1 functions as a key cofactor of PRC2 in mouse myotubes C2C12 under oxidative stress. Interestingly, chromatin isolation by RNA purification (ChIRP, an alternative assay to CHART [71]) performed with MALAT1 revealed the preferential binding of MALAT1 alone to intergenic and intronic regions ([27], see also Supplementary Figure S1a generated by the authors). Thus, we decided to perform our bioinformatic pipeline combining chromatin binding data from MALAT1 [27] and SUZ12 [72], generated by the Orlando group in this muscle cell model. Briefly, our pipeline shows 4758 peaks for MALAT1 and 3039 peaks for SUZ12, with 70 shared peaks between both molecules (Supplementary Table S4, Supplementary Figure S1b). The lower number of peaks compared to the breast cancer model may be explained by the lower expression of MALAT1 in non-cancer cells [13,14]. Interestingly, we see 37.14% of shared peaks localizing in gene promoters, plus 8.58% of signals belonging to the 5′-UTR (Supplementary Figure S1c). In sum, we confirmed that MALAT1 and SUZ12 selectively co-localize in regulatory regions in an independent cell type. We are currently conducting ChIP- and CHART-seq experiments in other human cancers where MALAT1 expression should be higher.

A caveat of our analysis is the strict approach of bedtools intersect (the tool used to identify the regions occupied by both molecules). Intersect delivers a peak where independent peaks overlap by one or more nucleotides [45]. This excludes from our analysis any gene where peaks may sit beside each other without overlapping, which is a possibility for two molecules binding to a gene. However, as our team is studying transcriptional regulation by a MALAT1–PRC2 complex, intersect’s strict approach is appropriate, narrowing the set of genes to those that exhibit overlap with high confidence. We are aware that our approach may be missing long-distance genomic interactions, due to higher-order interactions within the nucleus that go beyond gene promoter regions [73]. In fact, we have not included in our analyses the approximately 30% of MALAT1-SUZ12 overlapped peaks associated with introns and intergenic regions, which are contributing to higher-order spatial contacts or enhancer activity, among others.

## 5. Conclusions

In summary, by analyzing key datasets of genome-wide and transcriptomic studies performed in MCF7 cells, we identified a novel set of genes where a potential MALAT1–PRC2 functional collaboration can be now further tested at the molecular level. As epigenetic regulation has become a well-established driver for cancer development and progression [6], understanding the role of the PRC2–MALAT1 pair during the transcription of key genes in cancer cells will reveal alternative paths in the understanding and treatment of this disease.

## Figures and Tables

**Table 1:** A subset of genes that present an overlap of MALAT1 and SUZ12 in the gene promoter region (see also Figure 3d).

Gene Symbol	Full Gene Name	Related Functions	Ref.
*MEN1*	Menin	Part of CCKR signaling. Chromatin binding (component of a MLL/SET1 histone methyltransferase (HMT) complex). Negative regulator of cell cycle. Negative regulation of proliferation. Inactivation favors tumorigenesis of mammary cells.	[53]
*YWHAB*	Tyrosine 3-monooxygenase/tryptophan 5-monooxygenase activation protein beta	Part of EGF, FGF, and CCKR signaling. Stimulates cell growth.	[54]
*SENP3*	SUMO specific peptidase 3	Involved in processing of sumoylated proteins. High levels are associated with poor survival in breast cancer.	[55]
*MAPK15*	Mitogen-activated protein kinase 15	Part of multiple signaling pathways, including: PDGF, TGF-beta, EGF, FGF, IFG. Associated with apoptosis. Regulates autophagy, ciliogenesis, protein trafficking/secretion and genome integrity. Downregulation activates cell motility in breast cancer.	[56]
*CADM4*	Cell adhesion molecule 4	Cell–cell adhesion. Associated to wound healing. Associated with cell growth. Low expression associated with advanced breast cancer stages.	[57]
*TXNIP*	Thioredoxin interacting protein	Oxidative stress mediator. Transcriptional repressor. Tumor suppressor. Low expression in breast cancer.	[58]
*ETS2*	ETS proto-oncogene 2, transcription factor	Transcription factor. Regulator of telomerase for breast cancer cell survival. Promotes hTERT expression.	[59]
*RARG*	Retinoic acid receptor gamma	Receptor for retinoic acid. Downregulated in tumors. Silencing causes tamoxifen resistance in breast cancer.	[60]
*VEZF1*	Vascular endothelial zinc finger 1	Regulator of angiogenesis in cancer.	[61]
*KDM6B*	Lysine demethylase 6B	Demethylates lysine 27 of histone H3. Regulates HOX expression. Inhibits metastasis in breast cancer cells	[62]

## Data Availability

The scripts used in this study are available in the following GitHUb repository: https://github.com/CfierroR/rna_prc2_chromatin (accessed on 23 May 2023). ChIP-seq datasets used in this study can be downloaded from the GEO database (GSM2828862: https://www.ncbi.nlm.nih.gov/geo/query/acc.cgi?acc=GSM2828862 (accessed on 1 July 2021); GSM2828863: https://www.ncbi.nlm.nih.gov/geo/query/acc.cgi?acc=GSM2828863 (accessed on 1 July 2021); GSM2423179: https://www.ncbi.nlm.nih.gov/geo/query/acc.cgi?acc=GSM2423179 (accessed on 1 July 2021)). CHART-seq datasets used in this study can be downloaded from the GEO database (GSM1411209: https://www.ncbi.nlm.nih.gov/geo/query/acc.cgi?acc=GSM1411209 (accessed on 1 July 2021); GSM1411210: https://www.ncbi.nlm.nih.gov/geo/query/acc.cgi?acc=GSM1411210 (accessed on 1 July 2021); GSM1411212: https://www.ncbi.nlm.nih.gov/geo/query/acc.cgi?acc=GSM1411212 (accessed on 1 July 2021)). RNA-seq datasets used in this study can be downloaded from the GEO database (GSM1915041: https://www.ncbi.nlm.nih.gov/geo/query/acc.cgi?acc=GSM1915041 (accessed on 1 November 2022); GSM1172885: https://www.ncbi.nlm.nih.gov/geo/query/acc.cgi?acc=GSM1172885 (accessed on 1 November 2022)). Additional datasets supporting the conclusions of this article are included within the article and its supplementary files.

## Supplementary Materials

The following supporting information can be downloaded at https://www.mdpi.com/article/10.3390/cimb45060301/s1, Supplementary Table S1: Quality control data of the raw ChIP-seq SRA files used in this study from MCF7 breast cancer cells; Supplementary Table S2: The complete list of genes containing overlapped peaks of MALAT1 and SUZ12 in MCF7 cells; Supplementary Table S3: List of genes and their expression levels from GSE74232/GSM1915041/SRR2749729 and GSE48216/GSM1172885/SRR925723. TPM (Transcripts Per Kilobase Million) values are shown. Sheet 1: All genes from SRR2749729; Sheet 2: All genes from SRR925723; Sheet 3: Genes from SRR2749729 with MALAT1-PRC2 overlap in gene promoter region; Sheet 4: Genes from SRR925723 with MALAT1-PRC2 overlap in gene promoter region. Supplementary Table S4: The complete list of genes containing peaks of SUZ12, MALAT1 ad intersected peaks in C2C12 mouse myotubes. Sequence Read Archives (SRA) for MALAT1 binding experiments were recovered using the following run numbers: Replica 1: SRR12587947 (https://www.ncbi.nlm.nih.gov/sra/?term=SRR12587947 (accessed on 15 May 2023)); Input replica 1: SRR12587951 (https://www.ncbi.nlm.nih.gov/sra/?term=SRR12587951 (accessed on 15 May 2023)); Replica 2: SRR12587946 (https://www.ncbi.nlm.nih.gov/sra/?term=SRR12587946 (accessed on 15 May 2023)); Input replica 2: (https://www.ncbi.nlm.nih.gov/sra/?term=SRR12587950 (accessed on 15 May 2023)). SRA files for SUZ12 experiments were: Replica 1: ERR1458695 (https://www.ncbi.nlm.nih.gov/sra/?term=ERR1458695 (accessed on 15 May 2023)); Input replica 1: ERR1458693 (https://www.ncbi.nlm.nih.gov/sra/?term=ERR1458693 (accessed on 15 May 2023)). Supplementary Figure S1. Genomic distribution of MALAT1-SUZ12 overlapped peaks in the C2C12 mouse myotube cell line. (a) Genomic annotations for peaks were obtained using ChIPseeker. The percentage of peaks contained in each feature (colored rectangles) is shown for SUZ12 (left) and MALAT1 (right). (b) The number of peaks shared by SUZ12 and MALAT1 is shown with green background in the Venn diagram. (c) Genomic annotations for overlapped peaks were obtained using ChIPseeker. The percentage of peaks contained in each feature (colored rectangles) is shown.
