# Supplementary material for: Selective Concurrence of the Long Non-Coding RNA MALAT1 and the Polycomb Repressive Complex 2 to Promoter Regions of Active Genes in MCF7 Breast Cancer Cells
Source: Curr Issues Mol Biol. 2023 May 30;45(6):4735–48. doi: 10.3390/cimb45060301 (PMC10297253; doi:10.3390/cimb45060301)
Supplement: Supplementary file 1 [file cimb-45-00301-s001.zip › Supplementary Figure S1.zip!/Supplementary Figure S1.pdf]

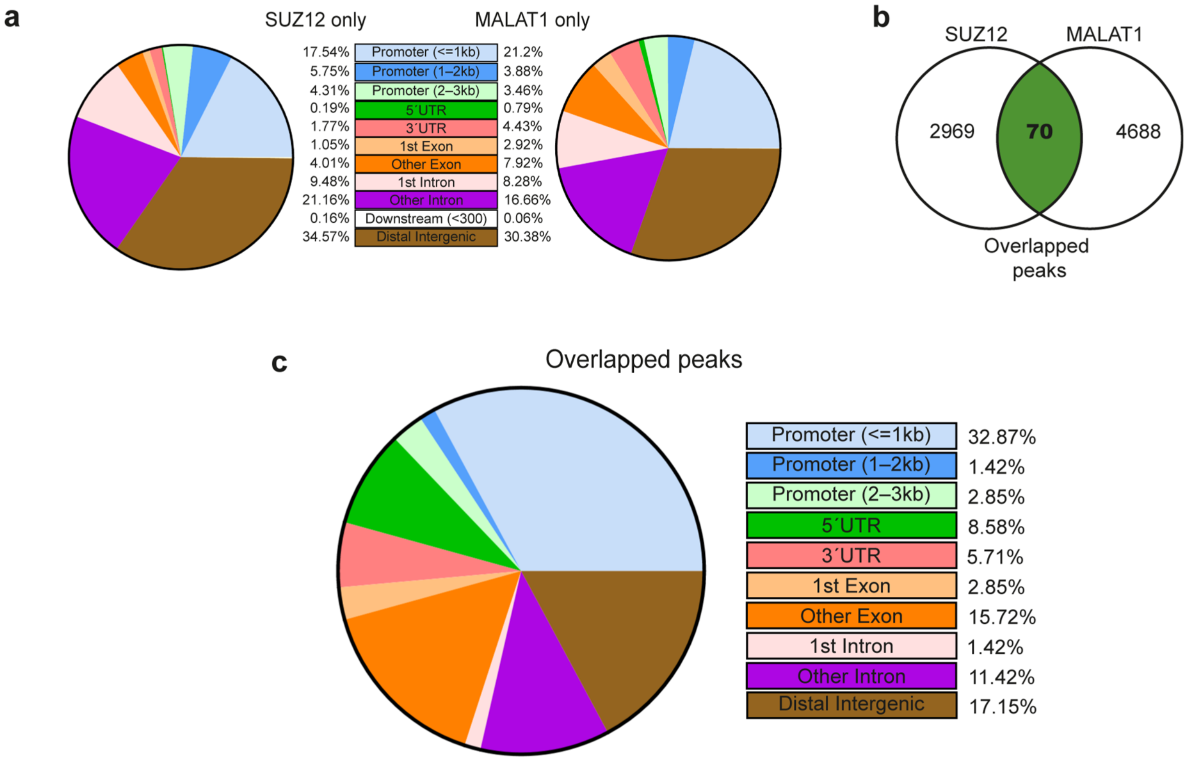

Supplementary Figure S1. Genomic distribution of MALAT1-SUZ12. overlapped peaks in the C2C12 mouse myotube cell line. (a) Genomic annotations for peaks were obtained using ChIPseeker. The percentage of peaks contained in each feature (colored rectangles) is shown for SUZ12 (left) and MALAT1 (right). (b) The number of peaks shared by SUZ12 and MALAT1 is shown with green background in the Venn diagram. (c) Genomic annotations for overlapped peaks were obtained using ChIPseeker. The percentage of peaks contained in each feature (colored rectangles) is shown.
